# Water-mediated green synthesis of PbS quantum dot and its glutathione and biotin conjugates for non-invasive live cell imaging

**DOI:** 10.1098/rsos.171614

**Published:** 2018-03-14

**Authors:** M. Vijaya Bharathi, Santanu Maiti, Bidisha Sarkar, Kaustab Ghosh, Priyankar Paira

**Affiliations:** 1 Department of Chemistry, School of Advanced Sciences, VIT University, Vellore 632014, Tamilnadu, India; 2 School of Electronics Engineering (SENSE), VIT University, Chennai Campus, Chennai, Tamilnadu, India

**Keywords:** PbS quantum dot, live cell imaging, green synthesis, glutathione, biotin, fluorescence microscopy

## Abstract

This study addresses the cellular uptake of nanomaterials in the field of bio-applications. In the present study, we have synthesized water-soluble lead sulfide quantum dot (PbS QD) with glutathione and 3-MPA (mercaptopropionic acid) as the stabilizing ligand using a green approach. 3-MPA-capped QDs were further modified with streptavidin and then bound to biotin because of its high conjugation efficiency. Labelling and bio-imaging of cells with these bio-conjugated QDs were evaluated. The bright red fluorescence from these types of QDs in HeLa cells makes these materials suitable for deep tissue imaging.

## 1. Introduction

Although green technology is advancing at a rapid rate, it does not meet the current demand in medicine and health field. The most challenging task is to diagnose diseases, including human immunodeficiency virus, Zika virus and cancer, using simple and cost-effective materials. With the advances in the field of nanoscience and nanotechnology, the above problem could be solved to some extent. Design and synthesis of bioconjugated quantum dots (QDs) is a hot field in current medical research because of their vast advantages over organic dyes [1].

Semiconductor QD has immeasurable applications in the medical field due to its various advantages such as broad excitation spectra, narrow emission bands, tunable emission peaks with respect to QD size, long fluorescence lifetimes, negligible photobleaching [2–4]. QD has high quantum yield, high molar extinction coefficients [3–5], large effective Stokes shift [6], the ability to conjugate to proteins and single-particle tracking regulating photochromic fluorescence resonance energy transfer [7]. Colloidal semiconductor PbS QD, a p-type semiconductor with a direct band gap of 0.37 eV at room temperature and vast exciton Bohr radius of 18 nm [8], offers tunable luminescence over visible and near-infrared (NIR) regions (400–2500 nm) by controlling the dot size [9–11]. PbS QD is one of the hot topics in the field of nanoscience and technology because of its wide application in biology [12] and solar energy field [13,14]. PbS QD for anti-HER2 bioconjugates shows a promising field for NIR-targeted molecular imaging with SK-BR-3 breast cancer cells [15]. The fluorescence peak in NIR IIa region is applied for deep tissue imaging because of negligible scattering of light [12]. Potential applications in bio-imaging using PbS and Ag_2_S QD in the second NIR window are reported in various aspects such as reduced toxicity [16] and KPL-4 cells with *in vivo* dual fluorescence [17]. RNase-A-capped PbS QD is applied for deep tissue imaging with ultra-low concentration with excellent fluorescence and reduced cytotoxicity [18]. A green strategy has been developed for the synthesis of whey-protein-capped PbS QD reusing unreacted precursors for bio-imaging application in the second NIR window [19]. Enhanced fluorescence of PbS QD by employing self-assembled molecular J-aggregates was also highlighted [20]. This type of QD has been applied for bioimaging and photoacoustic imaging [21,22].

Xu *et al.* compared the capping effect of PbS with those of benzodithiol, 1,2-ethanedithiol and mercaptopropionic acid (MPA) ligands, where PbS is passivated with MPA ligand, shortens the inter-dot separation, enhances the QD passivation and emission [23]. PbS QD capped with β-lactoglobulin was synthesized in an aqueous medium using microwave and examined on 293T cells in the second NIR optical window [24]. Kim *et al*. reported the cytotoxicity of bare PbS-MPA QD on human kidney cells (HEK 293) [25]. Hence, further surface modification of PbS-MPA QD is necessary for the diagnosis and selective delivery of a drug in cancer cells.

Sodium-dependent multivitamin transporter (SMVT; product of the *SLC5A6* gene) is an essential transmembrane protein responsible for translocation of vitamins and other essential cofactors such as biotin, pantothenic acid and lipoic acid [26]. As cancer cells have higher SMVT expression or higher biotin uptake capability than normal cells [26], the diagnosis of cancer and uptake of a drug in the cancer cells will be effective using biotin-conjugated QDs. While the affinity of streptavidin (SA) and biotin is high, the biotin conjugation to QD occurs via SA modification on QD [27]. Biotin conjugation to SA for biological imaging, sensing and target delivery has already been reported [28,29].

Water-soluble PbS QDs can be prepared in the aqueous phase using coating agents such as 1-thioglycerol/dithioglycerol [17], dihydrolipoic acid [30], l-cysteine [31], apoferritin [32] and luciferase [33]. Among these coated QDs, only those coated with 1-thioglycerol/dithioglycerol are emission tunable in the second NIR window.

However, PbS QDs coated with thiol compounds, such as 1-thioglycerol and dithioglycerol, are non-biocompatible and cytotoxic. Intracellular glutathione is the most abundant non-protein mono-thiol (1–10 mM) in cells that maintains intramolecular redox homeostasis through an equilibrium between its reduced thiol form (GSH) and its oxidized disulfide form [26,34,35]. To maintain the intramolecular redox homeostasis and to enhance the bio-imaging property of PbS QD, the antioxidant GSH modification on the surface of QD is highly warranted. We, therefore, considered GSH as a coating agent for the preparation of biocompatible second NIR-emitting PbS QDs. In this paper, we have synthesized PbS QD, capped with 3-MPA and the good biocompatible ligand GSH. In addition, surface modifications of MPA-coated QD were also performed with SA followed by conjugation with biotin. GSH-coated QDs can also be conjugated to biomolecules such as an antibody as they have two carboxyls and one primary amino group in their surface [36].

## 2. Results and discussion

**Figure 1.**
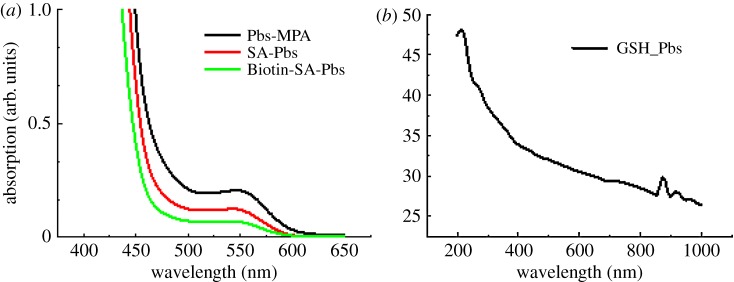
Absorption spectra of (*a*) PbS-MPA, (*b*) GSH-modified PbS quantum dots.

In continuation of our previous work [37], herein, we have used a sonication technique for the preparation of PbS-MPA and PbS-GSH QDs for further bioconjugation. Firstly, the sulfur and lead precursors were prepared by dissolving sodium sulfide and lead acetate trihydrate in deionized water. The synthesis was carried out separately for 3-MPA-capped and GSH-capped PbS QDs. Lead precursor was added to 3-MPA and GSH solutions by maintaining the required pH; after which sulfur precursor was added, turning both solutions darkish brown indicating the formation of PbS QD. The reaction was continued under sonication for appropriate time for the growth of PbS QD under nitrogen protection. After that, the reaction mixture was cooled down to room temperature, centrifuged and dried for further use. Figure 1*a* demonstrates the absorption spectra of 3-MPA-capped PbS QD with an absorption peak centred at 550 nm. No other visible absorption peaks can be seen which implies that the transitions, which can be attributed to 1se–1sh transitions individually, are occurring from other quantized states of the QD. More excitonic peaks in the absorption spectrum of PbS-GSH are attributed to the good crystalline quality of the nanoparticles. Broad absorption spectrum in the visible region indicates the fluorescence efficiency of PbS-GSH in the second NIR optical window which is very useful for deep tissue *in vivo* imaging (figure 1*b*). All these exciton transitions also demonstrate great quantum confinement impact as contrasted with bulk PbS. The absorption spectra started from the UV–visible region and extended up to 1800 nm (electronic supplementary material, figure S1), indicating the blue shift and significant quantum size. Nanocrystals exhibited threshold energy in the optical absorption measurement, which could be affected by blue shifting of the absorption edge with decreasing particle size. SA modification in 3-MPA-capped PbS QD occurred through EDC coupling reaction. SA-modified PbS QD was further conjugated with biotin. In the absorption spectra (figure 1*a*), it was observed that 3-MPA-capped PbS QD has a higher absorbance value at 550 nm than SA-modified PbS QD. Next orange graph is for biotin linked to PbS-SA QD shows slightly less intensity than PbS-SA QD, but both have the same wavelength of 550 nm [38].

**Figure 2.**
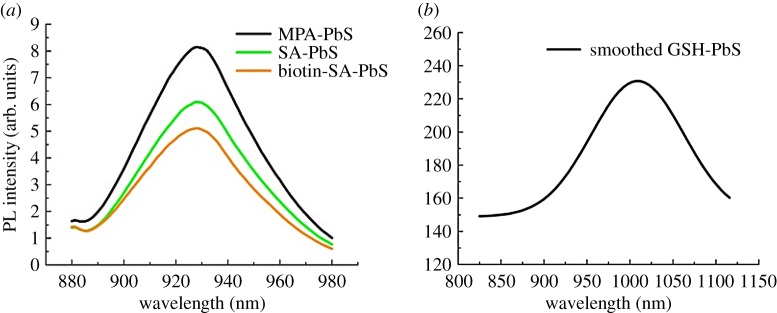
PL plot for (*a*) PbS-MPA, PbS-SA, PbS-SA-biotin, (*b*) PbS-GSH QD.

In the fluorescence (PL) spectra in figure 2*a*, the PbS-MPA QD emitted at 925 nm when excited at 550 nm, showing emission in the first NIR optical window which is suitable for bio-imaging. Chu *et al.* discovered that PL intensity increases, when the QD is synthesized in alkaline medium [39]. Herein, the PbS-MPA QD was synthesized in basic pH medium and then conjugated with SA using EDC coupling and further linked with biotin for efficient cellular uptake of the QD, having the emission at 925 nm (figure 2*a*). We also observed that PL spectra for PbS-SA and PbS-SA-biotin show fluorescence quenching. In figure 2*b*, emission of GSH-modified PbS was observed at 1010 nm when excited at 725 nm. The emission is in the second NIR-a optical window and the same can be extended up to the third NIR (1600–1870 nm) window with different excitation values, provided we have a source for excitation.

**Figure 3.**
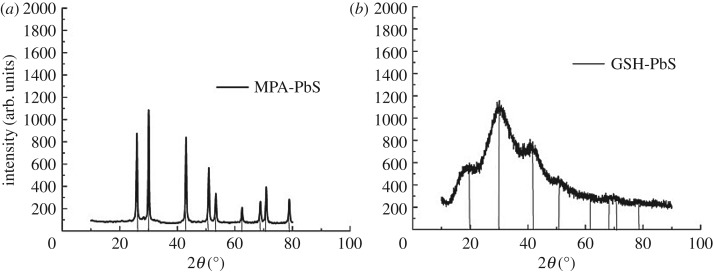
Powder X-ray diffraction pattern of (*a*) MPA-capped PbS QD, (*b*) GSH-capped PbS QD.

**Figure 4.**
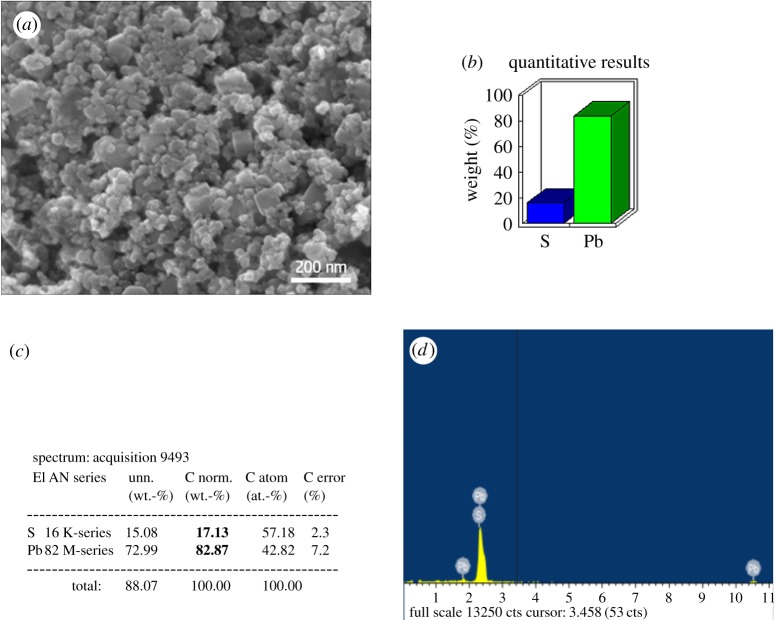
(*a*) SEM image, (*b*) graphical quantitative results in terms of %, (*c*) numerical elemental composition, (*d*) energy-dispersive spectrum of GSH-capped PbS quantum dots.

Figure 3 shows the powder X-ray diffraction (XRD) pattern for PbS QD, which confirms the crystalline structure of the PbS-MPA and PbS-GSH QDs. The broad peak at 2*θ* = 30.342° indicates the 1–10 nm nano size range of the QD. As revealed by the diffraction patterns, the peaks are at 25°, 30^o^, 42°, 51°, 53^o^, 62^o^, 68^o^, 71^o^ and 79^o^ corresponding to the (111), (200), (311), (222), (400), (331), (420), (422) and (511) plane of the PbS QD, confirming the face-centred cubic structure of the PbS QD as per the JCPDS file no. 77 0244. This result clearly indicates that it is purely the crystalline cubic structure of the QD. As per the XRD graph, PbS-GSH gives a broader and good crystalline structure when compared with PbS-MPA QD. Highly dense QDs of different shape and size in figure 4*a* clearly indicate the formation of GSH-capped PbS QD. The crystalline purity of GSH-capped PbS QD was further confirmed by energy-dispersive X-ray (EDX) spectrum (figure 4*d*).

**Figure 5.**
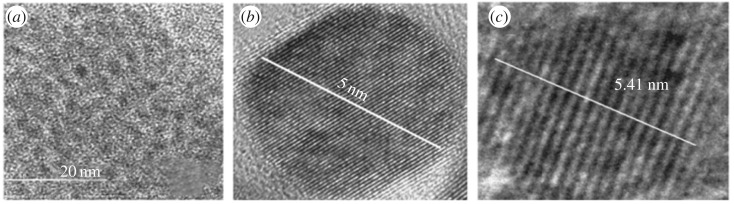
(*a*) TEM image and (*b*) HRTEM image of 3-MPA-capped PbS QD. (*c*) HRTEM image of GSH-capped PbS QD.

For cellular delivery of the nanomaterials, size is one of the main criteria which can be determined by transmission electron microscopy (TEM). High-resolution transmission electron microscopy (HRTEM) confirms the shape and size of the QD. Figure 5*a* shows the TEM image of PbS-MPA; the image indicates the particle is spherical, and has good homogeneity in the particle size distribution. Figure 5*b* shows the HRTEM image of the sample giving a clear indication of the size of 6 ± 0.5% nm and lattice plane in each QD. Figure 5*c* gives the shape and size of PbS-GSH QD of about 5 ± 0.5% nm which can be correlated with XRD data. Stability of the PbS QD was also well correlated with the zeta potential value (electronic supplementary material, figure S2).

Electronic supplementary material, figure S3, shows Fourier transform infrared (FTIR) spectra of surface-modified PbS QD in the range from 4000 cm^−1^ to 500 cm^−1^. In the electronic supplementary material, figure S3*a*, a broad peak at 3334 cm^−1^ is indicating the OH stretching vibration of the carboxyl acid group present in 3-MPA. The absence of peaks at 2665 cm^−1^ and 2565 cm^−1^ corresponding to S─H stretching of pure capping ligand shows the thiol-assisted capping around PbS QD. The sharp peaks at 1517 cm^−1^ indicate asymmetric stretch of COOH. The peak at 1389 cm^−1^ corresponds to C─H bending and another peak corresponds to 839 cm^−1^ indicating S─C vibrations. Electronic supplementary material, figure S3*b*, shows the FTIR spectrum for PbS-SA QD; amide bond peaks are exhibited at 3290 cm^−1^, 2978 cm^−1^, 1637 cm^−1^, 1548 cm^−1^, 1406 cm^−1^, 1027 cm^−1^, 926 cm^−1^ and 539 cm^−1^. Electronic supplementary material, figure S3*c*, shows the FTIR spectrum for GSH-capped PbS QD, where S─H vibration peak of the free GSH at 2524 cm^−1^ was not observed for PbS-GSH. This result confirms that GSH has coordinated to the surface of the PbS QD through sulfur. Free glutathione N─H vibrations at 3250 cm^−1^ were not observed indicating a change in hydrogen bonding. The peaks at 1405 cm^−1^ and 1640 cm^−1^ correspond to asymmetric and symmetric stretch of COOH.

Undoubtedly, cellular imaging is one of the essential practices in biology and medicine, and it is a vital method for cellular analysis, especially analysis of biological processes in cells. However, two major factors could control the usage of QDs in cellular imaging: one is cytotoxicity and the other is fluorescence stability. Fluorescence stability was evaluated by time-dependent fluorescence spectroscopy. It is shown in electronic supplementary material, figure S4, that with the extension of storage time, the fluorescence intensities of GSH-modified PbS QD and MPA-capped SA-biotin-modified PbS QD are increasing which sustain in their imaging efficiency. This is because of surface capping or surface modification [40]. The fluorescence stability of GSH-capped CdTe QD was highlighted by Yuan *et al.* [41]. Likewise, good photostability of CdSe QD was reported in a review article [42]. Long-term fluorescence retention of colloidal PbS QD was also described by Pichaandi *et al.* [43].

The cytotoxicity of as-prepared PbS QDs (PbS-SA-biotin and PbS-GSH), which was used to evaluate their biocompatibility, was studied using cervical cancer cell HeLa and human embryonic kidney cell HEK 293 as the model cell lines by the standard MTT method. Percentage of cell viability was measured from the absorption of different concentration of QD (0–800 µg ml^−1^)-treated cells at 570 nm. In the electronic supplementary material, figure S5, it was observed that MPA-capped SA-biotin-modified QD and GSH-capped PbS QD are not cytotoxic in both the cell lines which is favourable for cancer cell diagnosis. Log IC_50_ and corresponding IC_50_ of both the QD samples in HeLa and HEK 293 were observed in the range of 2.12–2.35 and 132–224 µg ml^−1^, respectively.

**Figure 6.**
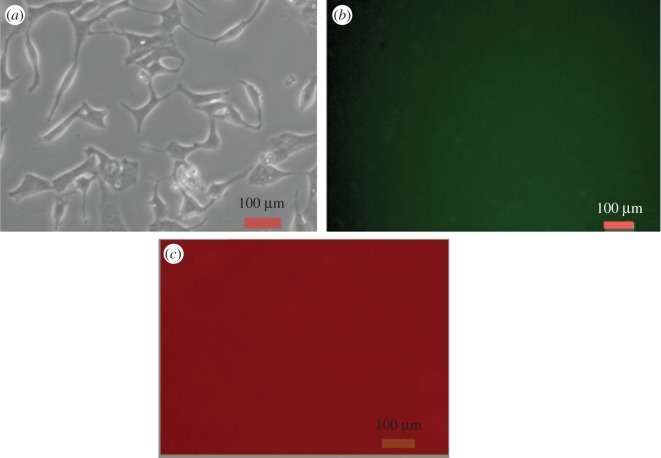
Fluorescence and bright field images of live HEK 293 normal kidney cells. (*a*) Bright field image of HEK 293 cells directly labelled by PbS-SA-biotin QD (3 × 10^−5^ M in PBS buffer) for 4 h. (*b*) Fluorescence image of HEK 293 cells directly labelled by PbS-SA-biotin QD (3 × 10^−5^ M in PBS buffer) for 4 h, green channel, scale bar 100 µm. (*c*) Fluorescence image of HEK 293 cells directly labelled by PbS-SA-biotin QD (3 × 10^−5^ M in PBS buffer) for 4 h, red channel, scale bar 100 µm.

**Figure 7.**
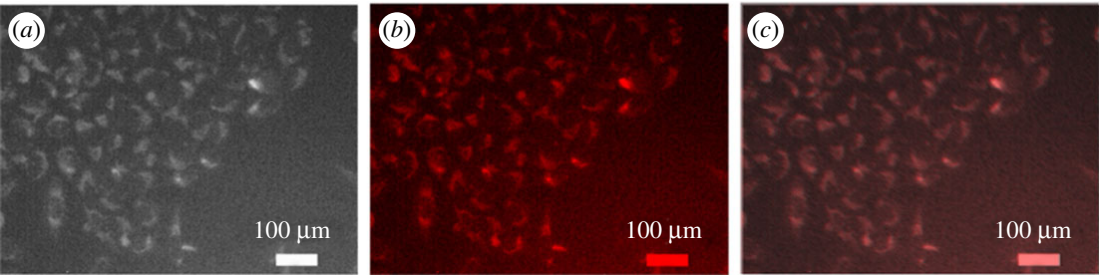
Fluorescence and bright field images of live HeLa cells. (*a*) Bright field image of HeLa cells directly labelled by PbS-SA-biotin (3 × 10^−5^ M in PBS buffer) for 4 h. (*b*) Fluorescence image of HeLa cells directly labelled by PbS-SA-biotin (3 × 10^−5^ M in PBS buffer) for 4 h, red channel. (*c*) Merged image of (*a*) and (*b*).

**Figure 8.**
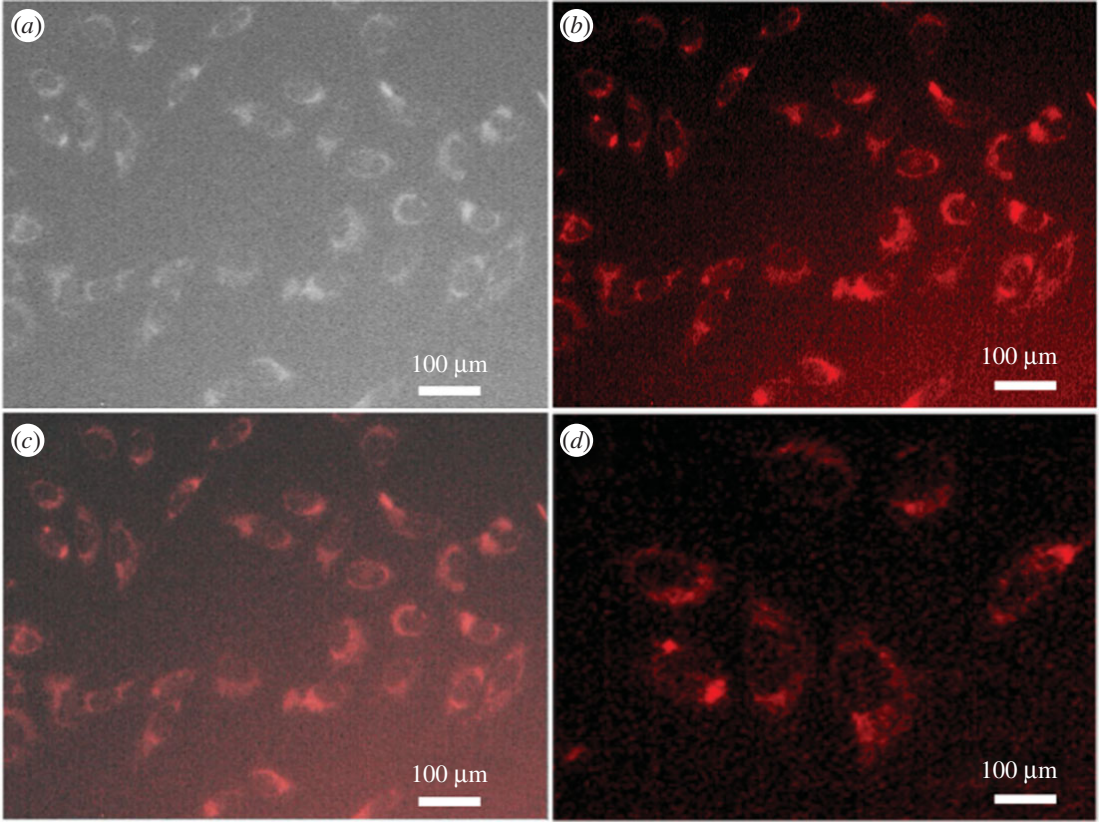
Fluorescence and bright field images of live HeLa cells. (*a*) Bright field image of HeLa cells directly labelled by PbS-GSH (3 × 10^−5^ M in PBS buffer) for 4 h. (*b*) Fluorescence images of HeLa cells directly labelled by PbS-GSH (3 × 10^−5^ M in PBS buffer) for 4 h, red channel. (*c*) Merged image of (*a*) and (*b*). (*d*) Zoom scan of (*b*).

To use these QDs in cellular imaging, we studied the cellular uptake of PbS-SA-biotin and PbS-GSH QD by cancerous HeLa cells and normal kidney HEK 293 cells. Cells were incubated with 3 × 10^−5^ M of QDs in cell culture with Dulbecco's modified Eagle medium (DMEM) for 4 h and monitored by using an Olympus fluorescence microscope. After 4 h of incubation with QDs, cancerous HeLa cells showed bright red fluorescence in the green filter of the microscope (figures 7 and 8). The aggregation of nanoparticles in the cytoplasm and nucleus is clearly visible, whereas no fluorescence was observed from normal kidney HEK 293 cells (figures 6–8). These results indicate the higher cellular uptake of biotin- and GSH-modified PbS QDs by the cancerous HeLa cell than normal kidney cell.

## 3. Conclusion

In summary, we have reported water-mediated green synthesis of biotin- and GSH-modified PbS QDs under sonication, which can open plausibility for applications in biological systems. The XRD and EDX spectra likewise revealed the formation of profoundly immaculate and crystalline QDs which can likewise be suitably used for optoelectronic and bioimaging application. Biotin- and GSH-modified PbS QDs exhibited excellent fluorescence stability and very low cytotoxicity in normal kidney cell and cancerous HeLa cell. Therefore, these types of QDs can be used for cellular tracking in the human body without affecting the normal cells. The bright red fluorescence from these types of surface-modified QDs in HeLa cells makes these materials suitable for deep tissue imaging.

## 4. Experimental section

### 4.1. Synthesis of glutathione-modified PbS quantum dot

GSH modified PbS was synthesized as per the following protocol. Firstly, 4 ml of 0.1 M l-glutathione was dissolved in 50 ml of Milli-Q water in a two-necked flask, stirred for 10 min under N_2_ protection by adjusting pH of the solution to 10 using 1N NaOH solution and then the solution was further stirred for another 10 min. One millilitre of 0.1 M lead precursor was added to the above mixture and stirred for another 10 min, after which 1 ml of 0.1 M sulfur precursor was added and immediately the pH was adjusted to 11 using NaOH solution and then stirred for 10 min with a Pb : S ratio of 1 : 1. The solution turned dark brown. To enhance the PL of the QD, another 1 ml of lead precursor was added to the above solution again by adjusting the pH to 11, the final ratio of GSH : Pb : S being 4 : 2 : 1. The final solution turned dark brown which confirmed the formation of GSH-modified PbS nanocrystals. For further growth of QDs, the solution was bath sonicated for 30 min at 50°C. After sonication, QDs were precipitated with an equivalent amount of 2-propanol, followed by resuspension in a minimal amount of ultrapure water. Excess salts were removed by repeating this procedure three times, and the purified QDs were dried overnight at room temperature in vacuum.

### 4.2. Materials

Lead acetate (99.9%), sodium sulfide, GSH, phosphate-buffered saline (PBS), MTT reagent, dimethylsulfoxide and trypsin were purchased from Sigma Aldrich. 3-MPA, sodium hydroxide and 2-isopropanol were purchased from Alfa Aesar. Streptavidin and biotin were purchased from Invitrogen. DMEM was purchased from Himedia. Cancer cell line (HeLa) and normal kidney cell line (HEK 293) were obtained from NCCS, Pune.

## Supplementary Material

Water mediated green synthesis of PbS Quantum Dot and its GSH and biotin conjugates for non-invasive live cell imaging†

## Data accessibility

A detailed experimental section can be found in the electronic supplementary material.
