# Supplementary material for: Water-mediated green synthesis of PbS quantum dot and its glutathione and biotin conjugates for non-invasive live cell imaging
Source: R Soc Open Sci. 2018 Mar 14;5(3):171614. doi: 10.1098/rsos.171614 (PMC5882692; doi:10.1098/rsos.171614)
Supplement: Water mediated green synthesis of PbS Quantum Dot and its GSH and biotin conjugates for non-invasive live cell imaging† [file rsos171614supp1.docx]

**Supporting information**

**Water mediated green synthesis of PbS Quantum Dot and its GSH and biotin conjugates for non-invasive live cell imaging**†

M. Vijaya Bharathi, ^[a, b, c]^ Santanu Maiti, ^[c]^ Bidisha Sarkar, ^[c]^ Kaustab Ghosh,^*[a, b]^ Priyankar Paira,^*[c]^

**Experimental Section**

**Chemicals**

The method used for synthesis of PbS quantum dots. Lead Acetate (99.9%), sodium sulphide, GSH was purchased from sigma Aldrich, 3-Mercaptoproponic acid, Sodium hydroxide, 2-isoproponal was purchased from Alfa Aesar and Streptavidin and biotin was purchased from Invitrogen. All chemicals used in the synthesis were of analytical grade used as received.

**Synthesis of 3-MPA modified PbS Quantum Dot**

1. **Synthesis PbS QD**

28µl (0.32mmole) of 3-Mercaptoproponic acid (MPA) was added to 40 ml of Mili Q water and stirred for 10 minutes by adjusting the pH of the solution to 9 using 1N ammonium hydroxide followed by continued stirring for another 5 min. Then 1ml of lead precursor solution was added to the above mixture and stirred for another 10 minutes, after which 1 ml of sulphur precursor was added and immediately adjusted the pH to 11 using NaOH solution. Then the mixture was stirred for 10 minutes with Pb:S ratio of 1:1 turning solution into dark brown. To enhance the PL of the QD another 1 ml of lead precursor was added to the above solution again by adjusting the pH to 11 using NaOH and stirred for 10 minutes, the final ratio of MPA:Pb:S was 4:2:1. The final solution turned into dark brown color confirming the formation of PbS QD nanocrystals. For further growth of QD the solution was bath sonicated for 30 min at 50^o^c. The complete process was carried out under N_2_ protection

1. **PbS-MPA QD Purification**

For further use, the MPA modified PbS sample was stored overnight at 4^o^C and was brought to powder form with precipitating the solution using the antisolvent ethanol and centrifuged at 4000 rpm for 10 minutes and then the supernatant was discarded, further the residue was dissolved in water and ethanol in the ratio of 1:3 and centrifuged, the process was continued until the pH is reached to 7. Finally, the sample was collected and dried overnight in vacuum at room temperature. The powder sample was collected and stored in a sample glass vial for further use.

**Surface modification of MPA modified PbS Quantum Dots with Streptavidin**

As prepared PbS QDs were functionalized by EDC coupling with the formation of primary peptide linkage between carboxyl group of PbS QD and amine group of streptavidin [10x]. The stock solutions of 20 mM *N*-ethyl-*N*-dimethylaminopropyl-carbodiimide (EDC) and 50 mM *N*-hydroxysuccinimide (NHS) was prepared in 0.1 M 2-(N-morpholino)ethanesulfonic acid (MES) buffer with optimal pH=6.5. The concentration of the stock solution of PbS QD and Streptavidin is 8 µM and 1mg/ml respectively. Initially, 1µM of PbS QD in 10mM borate buffer with pH=7.3 was added to 12 mM of EDC and 5mM of NHS. The mixture was stirred for 30 minutes at room temperature and then 4 µM of SA solution was added to the above mixture, further incubated for two hours at room temperature, with QD to SA ratio 1:4, as the conjugation efficiency is high for this ratio.**^1^**

**Conjugation of QD-SA with Biotin**

20 µl of the 10 µM of biotin was mixed with 4 µl of 2 µM QD-SA solutions and 2.5 µl of 10X PBS buffer, and final reaction volume was top up with water to 50 µl. The conjugation reactions were performed in 10XPBS buffer for 1 hour at room temperature and mixed at 800 rpm

**Synthesis of GSH modified PbS Quantum Dot**

GSH modified PbS was synthesized as per the below protocol given. First 4 ml of L-Glutathione of 0.1 M was dissolved in 50 ml of mili Q water in a two necked flask, stirred for 10 min under N_2_ protection by adjusting PH of the solution to 10 using 1N NaOH solution and again the solution was stirred for another 10 minutes. 1ml of 0.1 M lead precursor was added to the above mixture and stirred for another 10 minutes, after which 1ml of 0.1M sulphur precursor was added and immediately the pH was adjusted to 11 using NaOH solution and stirred for 10 minutes with Pb:S ratio of 1:1. The solution was turned into dark brown, to enhance the PL of the QD another 1ml of lead precursor was added to the above solution again by adjusting the pH to 11, the final ratio of GSH:Pb:S was 4:2:1. The final solution turned into dark brown color which confirmed the formation of GSH modified PbS nanocrystals. For further growth of QD the solution was bath sonicated for 30 minutes at 50^o^ c. After sonication, QDs were precipitated with an equivalent amount of 2-propanol, followed by resuspension in a minimal amount of ultrapure water. Excess salts were removed by repeating this procedure three times, and the purified QDs were dried overnight at room temperature in vacuum.

***In vitro* cytotoxic activities (MTT assay):** *In vitro* cytotoxicity was determined using the standard MTT assay. **^2^** The MTT proliferation assay is based on the reduction of the yellow MTT tetrazolium salt (3-[4,5-dimethylthiazol-2-yl]-2,5diphenyltetrazolium bromide) by mitochondrial dehydrogenases to form a blue MTT formazan in viable cells. 3-MPA capped PbS QD with streptavidin-biotin modification and GSH capped PbS QD were practiced aforementioned to the experiment by dissolving in buffer followed by serial dilution with medium. One cancer cell lines i.e. human Epitheloid Cervix Carcinoma (HeLa), and one normal Human embryonic kidney cells (HEK-293) were used in the assay. Approximately 1 × 10^4^ cells/ml in each well for all the cell lines were cultured in 100 μL of a growth medium in 96-well plates and incubated at 37 °C under a 5% CO_2_ atmosphere. The cells were then treated with different concentrations of the PbS (0-800 µg/ml) in the volume of 100 µL /well. Cells in the control wells accepted the same volume of medium containing 0.1% DMSO. After 24 h, the medium was discarded and cell cultures were incubated with 100 µl MTT reagent (1 mg/ml 1 mg/mL in PBS buffer with pH 7.4) for 5 h at 37^o^ C. After removing the solution, the cells were lysed by adding 200 μL methyl sulfoxide. Then the suspension was placed on microvibrator for 10 min and subsequently the absorbance was recorded by the ELISA reader at λ = 570 nm. The experiment was also performed in triplicate. The data were expressed as the growth inhibition percentage calculated according to the equation: % Cell viability = [OD_sample_-OD_blank_/OD_control_-OD_blank_] x 100, where OD_sample_ is the measured absorbance in wells containing samples, OD_control_ is the absorbance measured for cells with a medium and a vehicle and OD_blank_ is the absorbance measured for blank well (no cells). Dose response curve was fitted in origin 6.1 software and IC_50_ was calculated.

**METHODOLOGY FOR CANCER CELL FLUROSCENCE IMAGING:**

HeLa cell line from NCCS was used for this study. Cellular uptaking study of the drugs was carried out in 6 well plates. Cultured cells with 80% confluence were taken and trypsinisation was done by using 1-2ml of 1X trypsin. Then it was transferred to fresh 15 ml falcon tube and centrifuged at 2000 rpm for 5 minutes. DMEM fresh media (80µl) was added to the pellet formed at the bottom of the tube and the cells were seeded in 6 well plates. Then Sample B (3X10^-5^M in PBS buffer) was added to well plates. After incubated for 4 h at 37 °C, all the wells in 6 well plates washed twice with PBS buffer (pH 7.4). The glass slides as the fluorescence images were prepared using the cover glass of the 6-well plates. Then, the fluorescence images were recorded with an Olympus Fluorescence microscope with both the excitation filter (green channel: 460-490 nm excitation and red channel: 480-550 nm excitations).

**Figure S1: Absorption graph for GSH_PbS for UV-Vis region to 3rd optical window upto 1800 nm.**


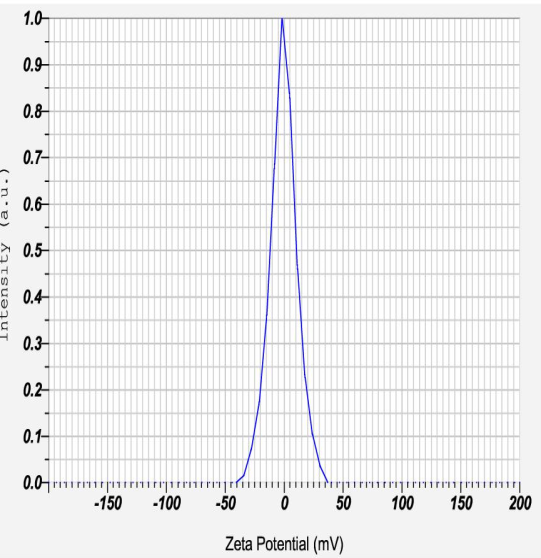

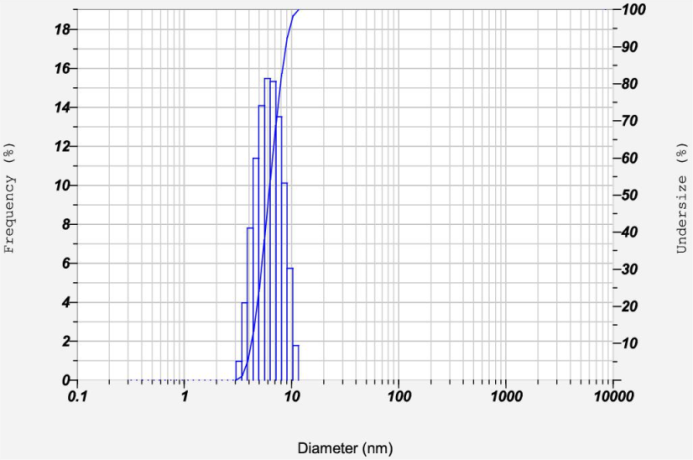


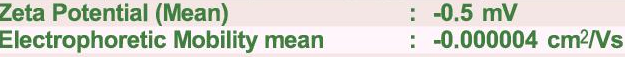


(a) (b)

**Figure S2: (a) Graph showing the Zeta potential value of -0.5mV (b) Graph shows the variation of QD size 1 nm to 5 nm in diameter.**


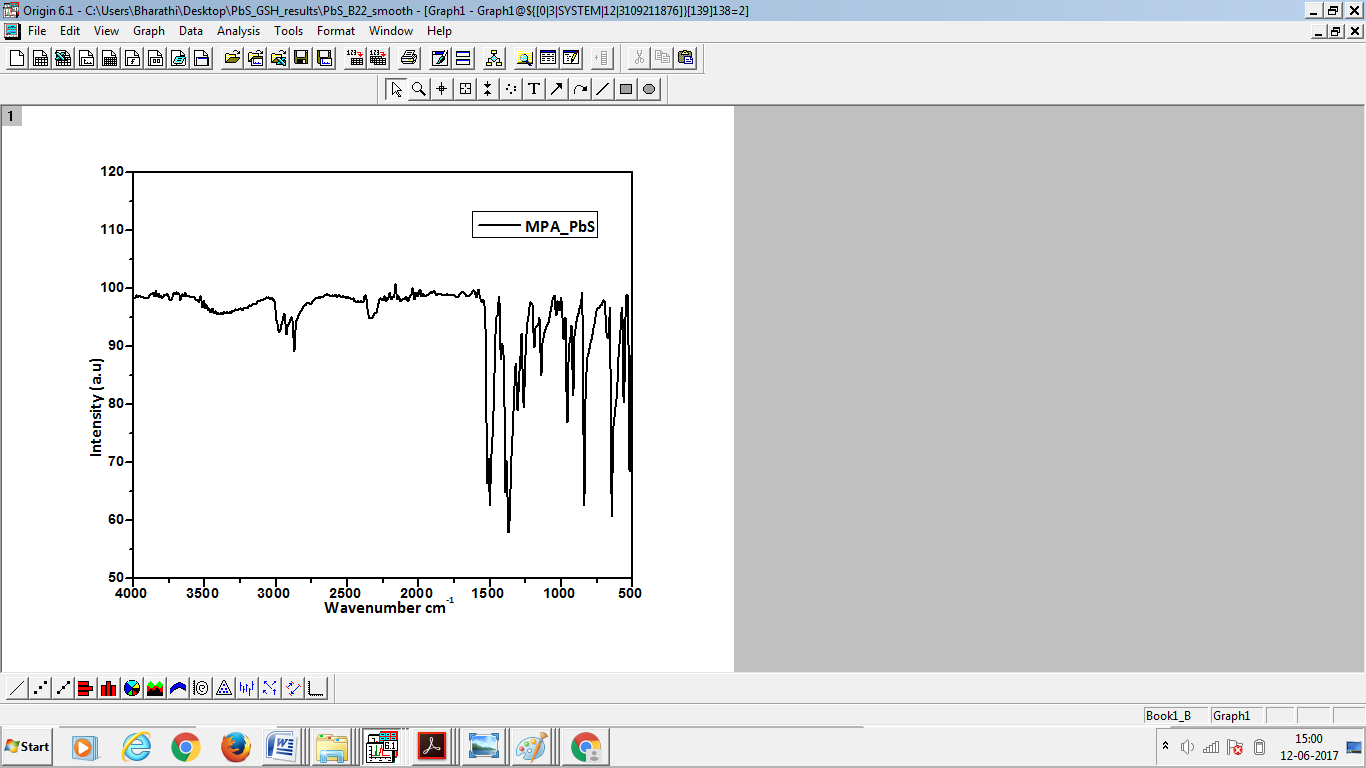


(a)


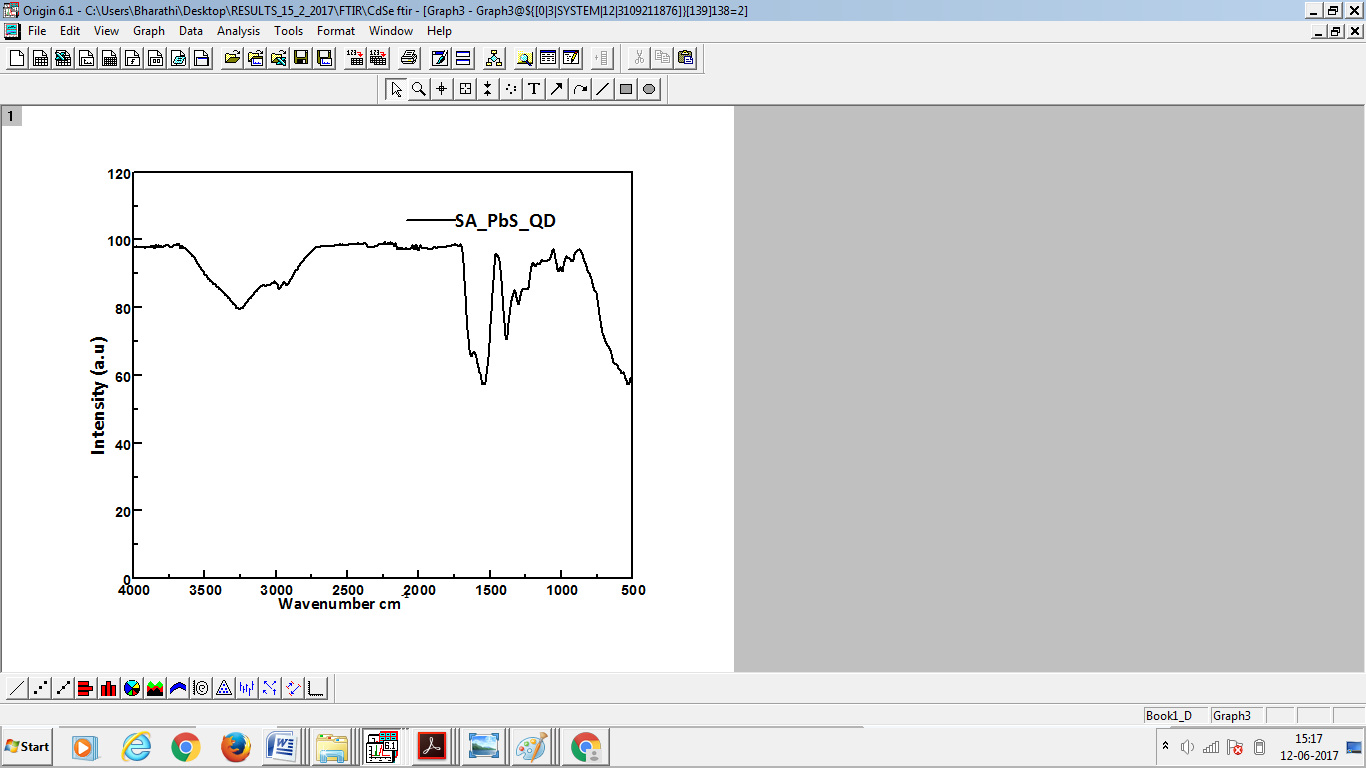


(b)


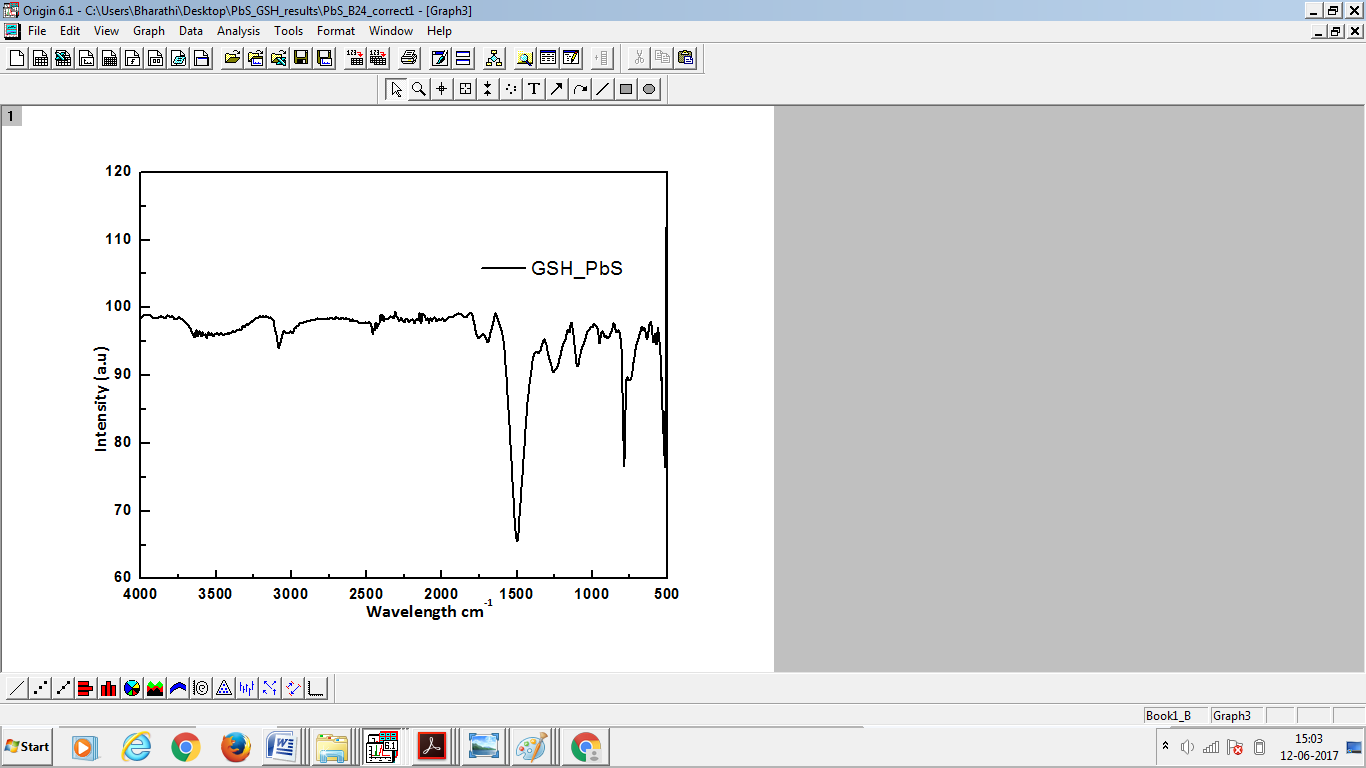


(c)

**Figure S3: FT-IR graph for (a) MPA_PbS, (b) Surface modified with SA_PbS, (c) GSH_PbS Quantum Dot.**

**(a)**

**(b)**

**Figure S4 Fluorescence stability of (a) GSH capped PbS QD (b) MPA capped SA-Biotin modified PbS QD**

**
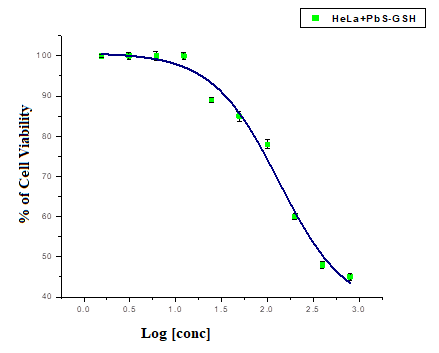
**

**(a)**

**
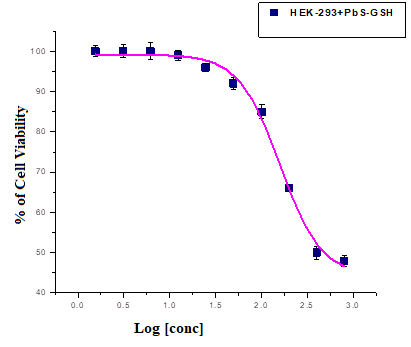
**

**(b)**


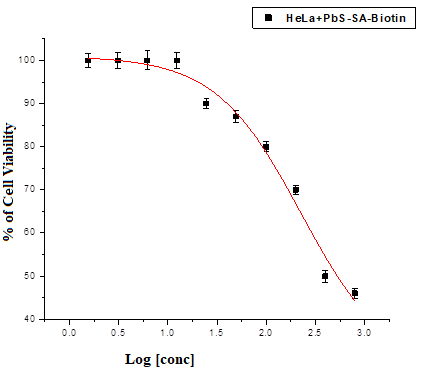


**(c)**

**
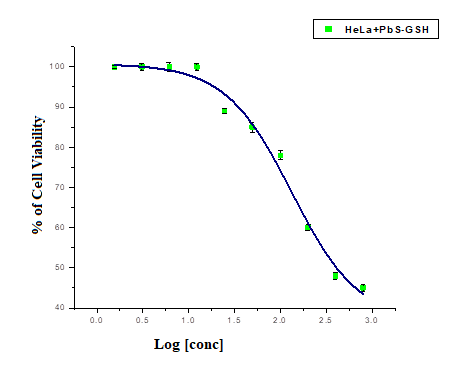
**

**(d)**

**Figure S5. The influence of PbS-SA-Biotin QD on the cell viability of (a) HEK-293 cell line (c) HeLa cell line; the influence of PbS-GSH QD on the cell viability of (b) HEK-293 cell line (d) HeLa cell line**

**References**

1. G. H. Au, W. Y. Shih, W. H. Shih, *Analyst*, **2013**, *138*, 7316.
2. (a) T. Mossman, *J. Immunol. Methods*, **1983**, *65*, 55-63.; (b) S. Saeed, N. Rashid, P. G. Jones, M. Ali and R. Hussain, *Eur. J. Med. Chem.*, **2010**, *45*, 1323-1331.
